# Ischemic Gangrene of the Glans following Penile Prosthesis Implantation

**DOI:** 10.1155/2013/323574

**Published:** 2013-07-11

**Authors:** Borja García Gómez, Javier Romero Otero, Laura Díez Sicilia, Estibaliz Jiménez Alcaide, Eduardo García-Cruz, Alfredo Rodríguez Antolín

**Affiliations:** ^1^Urology Department, Hospital Universitario 12 Octubre, Avenida. De Córdoba s/n, 28041 Madrid, Spain; ^2^Red Española de Investigación en la Salud del Hombre (REISHO), 08037 Barcelona, Spain; ^3^Urology Department, Hospital Clínic Barcelona, Villarroel 170, 08037 Barcelona, Spain

## Abstract

The development of ischemic gangrene of the penis following implantation of prosthesis is unusual, and very few cases are available in the literature. As a result, no established treatment protocol is available. We report our experience within a case of gangrene of the glans following implantation of a three-component prosthesis. We present a 53-year-old male, smoker with diabetes and hypercholesterolemia, who underwent surgery for the insertion of a penile prosthesis with 3 components to correct his erectile dysfunction and severe Peyronie's disease. The procedure was carried out without incidents. During the postoperative period, the patient began to complain from penile and perineal pain. He developed avascular necrosis of the glans. The necrosed area was excised. Four weeks later, he developed fever and perineal pain arriving to the emergency room with the prosthesis extruding through the glans. He had emergency surgery to remove the prosthesis plus surgical lavage and was prescribed broad-spectrum antibiotic therapy. Four weeks later, the penis was completely revascularized and reepithelialized. Ischemic gangrene following penile prosthesis implantation takes place in patients with poor peripheral vascularisation. Diabetes mellitus has been the common denominator to all of the reported cases.

## 1. Introduction

For more than twenty years, penile prosthesis (PP) has been accepted worldwide as a treatment for erectile dysfunction (ED). The implant of these devices is more extended year after year, and accordingly, the experience acquired is increasing. There are several common complications regarded to penile prosthesis, including pain, mechanical failure, poor placement, erosion, and infection [1]. Most of them are well documented and therefore include a standardized treatment. However, the development of ischemic gangrene after PP implantation is very uncommon and very few cases have been reported [1–5], making it difficult to establish the real incidence of the issue. Furthermore, as very few cases have been reported, no established treatment protocol has been recognized yet. Although conservative management has been described in few cases, the great majority require the removal of the PP [3]. Our aim is to describe our experience with a case of ischaemic gangrene of the distal penis after implantation of a PP in order to describe our intervention.

## 2. Case Presentation

A 53-year-old male was derived to our clinic complaining of erectile dysfunction. His past medical history included being a current smoker of 20 cigarettes/day for 30 years, noninsulin-dependent *diabetes mellitus* for 14 years, hypercholesterolemia, and gastroesophageal reflux. The patient had undergone a vasectomy 20 years ago. Although blood glucose was within normal limits in preoperative tests, his previous glycosylated haemoglobin was 10.4%. Written informed consent from the patient was obtained for the case report to be published.

The patient presented at our clinic with severe ED (International Index of Erectile Function: 7 points) and a dorsal Peyronie's disease (PD) (80°) for more than two years, disabling the patient for intercourse. The physical examination revealed a calcified plaque of 4 × 2 cm in the dorsal surface of the penis. Several options were discussed with the patient, and finally PP implantation was chosen in order to treat both pathologies, correcting ED and the penile deviation.

During surgery, cefazolin was administered as antibiotic prophylaxis as part of our surgical protocol, and the operation site was thoroughly cleaned.

**Figure 1:**
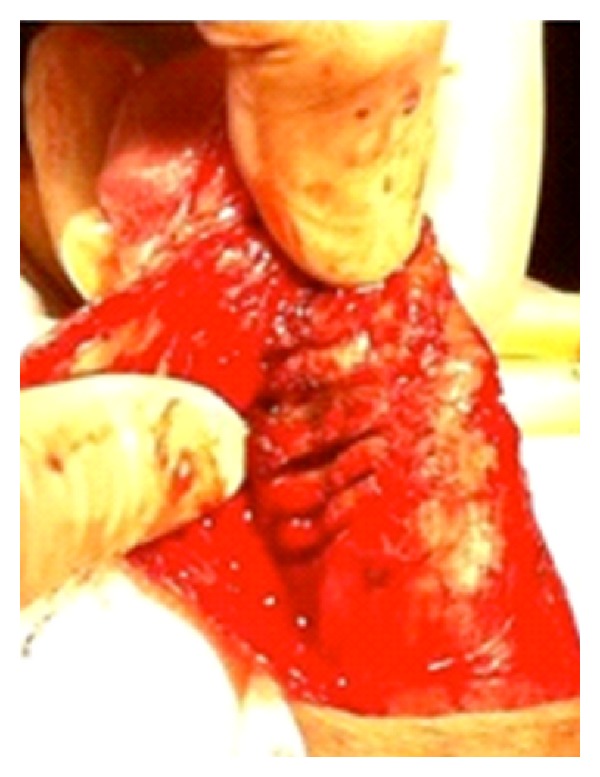
Incision in the calcified 4 × 2 cm plaque.

Usually, one PP is implanted to treat both PD and ED, fill both cylinders, and force modelling of the penis. However, in this case, due to the severity of the PD and the hardness of the plaque, an incision of the PD plaque was firstly performed and later on forced. In order to perform this, the usual surgical technique for PD was used: an incision was made at the coronal sulcus, the penile shaft denuded, and the neurovascular bundle carefully dissected with bipolar cautery (starting from urethral insertion). Several incisions were made to the plaque (Figure 1), avoiding entering the corpus cavernosum.

**Figure 2:**
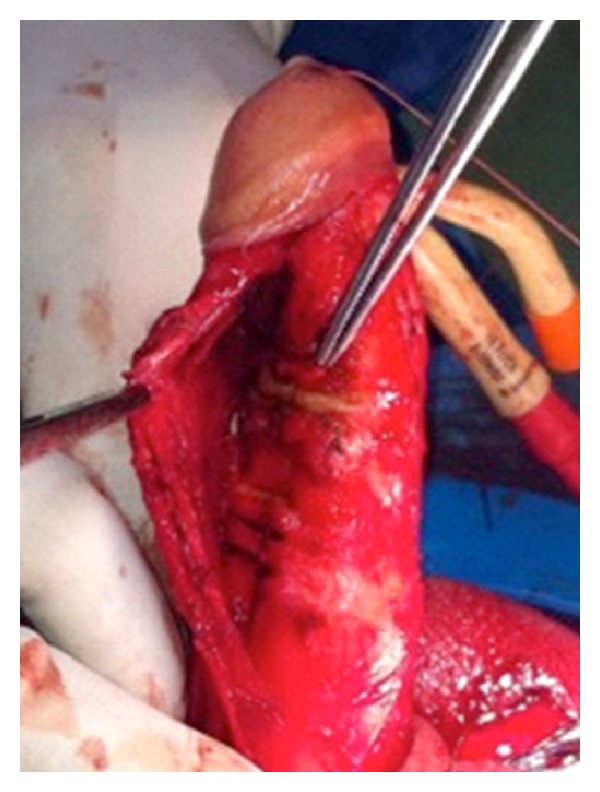
Correction of the deviation following the penile prosthesis implantation.

Next, the PP was implanted following the standardized steps: through a penoscrotal approach, a 15 cm CX700 InhibiZone prosthesis (AMS) was implanted adding 3 cm extenders. A forced modelling of the penis was then performed. Complete correction of the deformity and proper functioning of the implant were confirmed (Figure 2). A suction drain was left in situ, and a noncompressive elastic penile bandage dressing applied as per the usual postoperative care. The initial postoperative period went on without incidents, and the patient was discharged on the second day after surgery with antibiotic therapy: amoxicillin-clavulanic acid 875/125 7 days.

**Figure 3:**
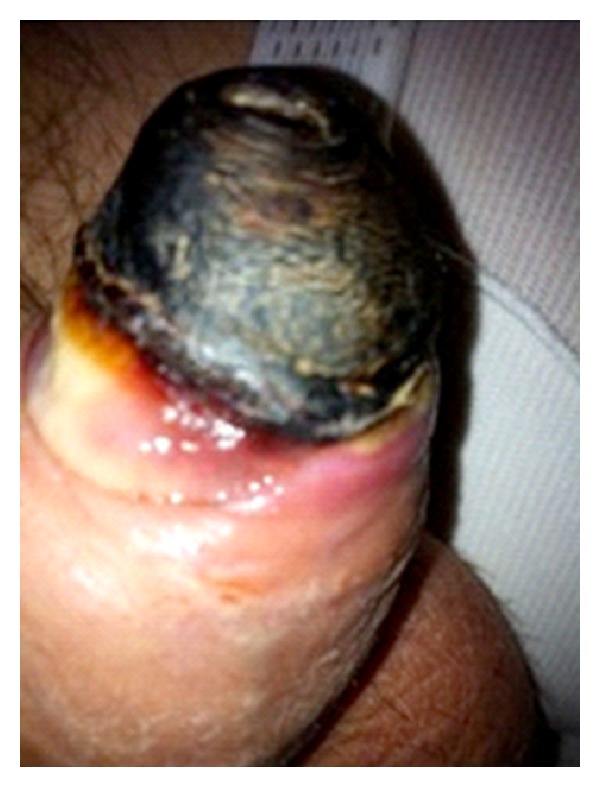
Avascular necrosis of the glans.

Seven days after surgery, the patient returned to our clinic complaining of penile and perineal pain, for which he had required nonsteroidal anti-inflammatories. On physical exam, both the wound and penis appeared to be fine, except for the distal penis, which seemed to be very pale. At that moment, the decision of watchful waiting was made. Twenty days after surgery, patient came to the clinic once more due to the gradual appearance of a painless blackened area at the distal end of the glans. Patient had no fever or other signs of infection. A Doppler ultrasound of the penis was performed, showing no vascularization of the distal penis. The patient underwent Tadalafil 5 mg/daily and massage of the penis due to the suspicion of an ischemic procedure. Unfortunately, forty-eight hours later an ischaemic necrosis of the glans was diagnosed (Figure 3). Immediate surgery to excise the necrotic area was performed, as well as debridement of the ischemic tissue. Four days after the emergency surgery, the patient was discharged. He attended the clinic for wound care, and antibiotic therapy was resumed with amoxicillin-clavulanic acid 875/125 every 8 hours. At this point, no sign of infection was observed and therefore making the patient candidate for conservative care.

**Figure 4:**
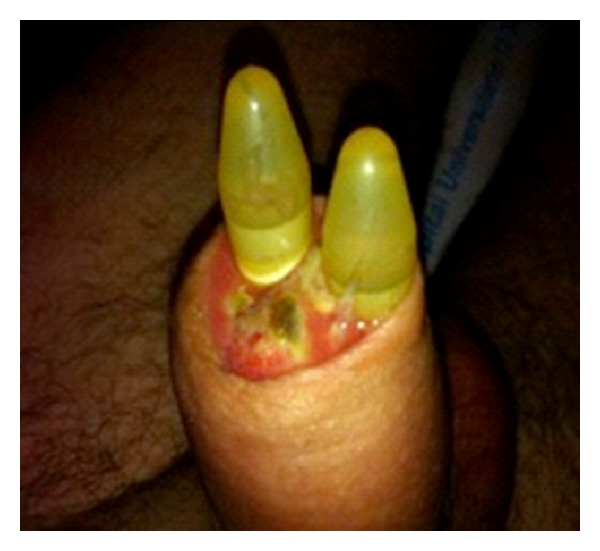
Extrusion of the penile prosthesis through the glans.

Despite a favourable initial response of the patient, four weeks later, he was attended in the emergency room complaining of high fever (39°C), perineal pain, and extrusion of the prosthesis through the glans (Figure 4).

**Figure 5:**
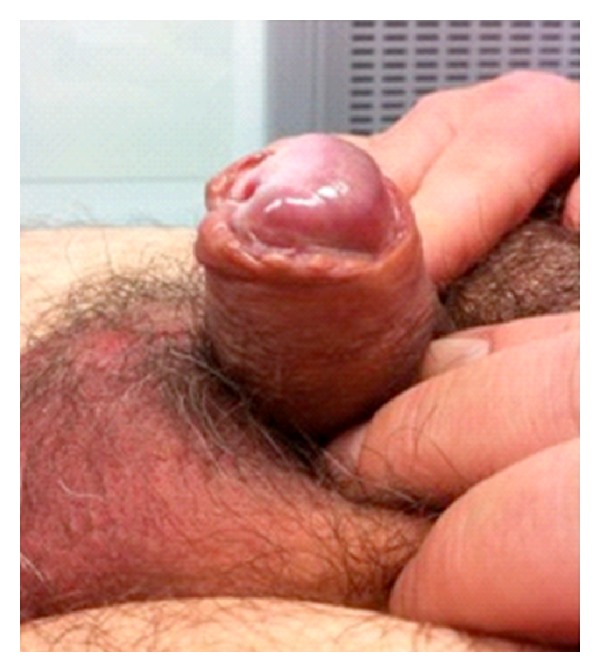
At the end physical examination, the penis appeared well vascularized once again and re-epithelialized.

The patient underwent surgery (third time) to remove the PP, excise the necrotic tissue, debride the ischemic/infected tissue, and wash out and drain the purulent material. A urinary catheter was left in situ, and he was treated during four weeks with IV ceftriaxone, based on an antibiogram from cultures of the purulent material and PP (positive for *Klebsiella pneumoniae* and *Morganella morganii*). During that time, the patient made good progress, both clinically and in terms of blood test results, remaining afebrile and with disappearance of the perineal pain. A pelvic-perineal magnetic resonance imaging (MRI) was performed four weeks later, which showed no signs of collections or other complications. On physical examination, the penis appeared well vascularised once again and reepithelialized (Figure 5).

## 3. Discussion

Penile prosthesis is a well-established treatment for erectile dysfunction [6]. Some of the most common complications include pain, mechanical failure, poor placement, erosion, and infection [1]. However, infection is the most feared complication, and the rate within the first prosthesis ranges from 1.61% to 9.9% [7], despite its drastic fall since the introduction of antibiotic-coated devices [8, 9].

Gangrene refers to tissue necrosis. There are various forms of necrosis, however, two are the most common, dry, caused by a lack of blood supply to the tissue, and wet, secondary to infection, usually bacterial. Differentiating between these two entities may not be as easy as it seems, as it is always possible that a tissue that has suffered ischaemic necrosis becomes superinfected. Broad-spectrum antibiotic prophylaxis would therefore seem advisable in cases of purely ischaemic necrosis [10].

Patients with acute or chronic ischemia are those at the highest risk of developing gangrene of the penis. This condition is associated with priapism, intense external pressure on the penis (clothing, suction devices), Wegener's granulomatosis, septic emboli in IV drug users, perivascular invasion of the penis by a tumour, penile surgery, and, most commonly, diabetes mellitus (DM) [11–15]. Several factors can act simultaneously causing gangrene of the penis. Moreover, wet gangrene, in which a bacterial agent causes an infection that necroses the tissue, can be more common in immunocompromised patients such as transplant recipients, HIV-positive, or DM [2, 16].

Ischemic gangrene of the penis associated with implantation of penile implants is extremely uncommon. The few cases described up to date have occurred within all types of penile prostheses, either malleable or with two or three components. Apparently, no higher incidence is observed with any device available on the market [1–3, 5]. Diabetes mellitus is the only risk factor associated with all cases, as it occurred in the present case, being the only risk factor that presented the patient. However, to date, nobody has related an increased level of glycosylated haemoglobin to a higher incidence of dry gangrene. A study by Minervini et al [17] found no correlation between increased levels of glycosylated haemoglobin (>11.5%) and prosthesis infections in diabetic patients. On the other hand, prosthesis infection has been reported to occur in poorly controlled patients with long-term diabetes [18]. We believe that these poorly controlled patients with altered glycaemia were a determining factor in the initial ischemia and the subsequent worsening of the condition, which finally developed superinfection.

Regarding the dissection of the neurovascular bundle and plaque incision and its possible contribution to the ischemia procedure, we still harbour several doubts. However, we have been using the same surgical technique to correct PD, making an incision to the plaque or, even more aggressive, excising the plaque, and no ischemic complications have been observed.

In all cases of ischemic penile gangrene published to date, surgical debridement of the necrosed area was necessary, and in all except one, the prosthesis had to be removed. The case we present is that of a young man with DM as risk factor, who simultaneously underwent an implant of penile prosthesis and correction of his severe Peyronie's disease, with excellent cosmetic and functional results. Consequently, we decided to exhaust all the conservative treatment options before resorting to removal of the penile implant when the patient presented with a possible ischemia event, and initially, results were satisfactory. Yildirim et al [1] suggested that in the event of an ischaemic lesion developing, the necrosed area could be debrided immediately to prevent bacteria from invading the tissue and thus avoiding gangrene but without the immediate removal of the prosthesis. We therefore went ahead with the conservative treatment, with the full agreement of the patient, aware of the fact that if our conservative management failed, more extensive amputation would probably be necessary to prevent the progression of the disease [19]. Unfortunately, the patient suffered a superinfection, which led to wet gangrene with massive tissue destruction and the subsequent extrusion of the prosthesis. In line with routine clinical practice, we decided to remove the prosthesis, obtaining good outcomes.

Our case provides support to the approach that predominates in the literature of early removal of the prosthesis in cases of ischaemic gangrene and associated diabetes mellitus.

When the gangrene presents as infective from the start, with fever and other local signs of infection, the treatment of choice would be the immediate removal of the prosthesis accompanied by debriding of the areas affected by necrosis and, if necessary, including partial amputation. In certain cases, rescue treatment can be possible with immediate replacement of the prosthesis [20]. However, when there is perforation and massive destruction of tissue as it was in the present case, this option is contraindicated.

Ischaemic gangrene of the penis is extremely uncommon. This event occurs almost exclusively in diabetic patients who, by definition, present poor peripheral vascularisation. It has been reported in the literature that if signs of active infection do not accompany ischemic necrosis, conservative management can be attempted. However, in the present case, the conservative treatment failed, making the removal of the penile prosthesis necessary. When wet gangrene is suspected, the treatment choice should be immediate removal of the prosthesis and debriding of the damaged area, including partial amputation of the penis if necessary.
